# Evolutionary Sweeps of Subviral Parasites and Their Phage Host Bring Unique Parasite Variants and Disappearance of a Phage CRISPR-Cas System

**DOI:** 10.1128/mbio.03088-21

**Published:** 2022-02-15

**Authors:** Angus Angermeyer, Stephanie G. Hays, Maria H. T. Nguyen, Fatema-tuz Johura, Marzia Sultana, Munirul Alam, Kimberley D. Seed

**Affiliations:** a Department of Plant and Microbial Biology, University of California, Berkeley, Berkeley, California, USA; b icddr,b, Dhaka, Bangladesh; c Chan Zuckerberg Biohub, San Francisco, California, USA; University of Pittsburgh

**Keywords:** CRISPR-Cas, *Vibrio cholerae*, bacteriophages, cholera, evolution

## Abstract

Vibrio cholerae is a significant threat to global public health in part due to its propensity for large-scale evolutionary sweeps where lineages emerge and are replaced. These sweeps may originate from the Bay of Bengal, where bacteriophage predation and the evolution of antiphage counterdefenses is a recurring theme. The bacteriophage ICP1 is a key predator of epidemic V. cholerae and is notable for acquiring a CRISPR-Cas system to combat PLE, a defensive subviral parasite encoded by its V. cholerae host. Here, we describe the discovery of four previously unknown PLE variants through a retrospective analysis of >3,000 publicly available sequences as well as one additional variant (PLE10) from recent surveillance of cholera patients in Bangladesh. In recent sampling we also observed a lineage sweep of PLE-negative V. cholerae occurring within the patient population in under a year. This shift coincided with a loss of ICP1’s CRISPR-Cas system in favor of a previously prevalent PLE-targeting endonuclease called Odn. Interestingly, PLE10 was resistant to ICP1-encoded Odn, yet it was not found in any recent V. cholerae strains. We also identified isolates from within individual patient samples that revealed both mixed PLE(+)/PLE(−) V. cholerae populations and ICP1 strains possessing CRISPR-Cas or Odn with evidence of *in situ* recombination. These findings reinforce our understanding of the successive nature of V. cholerae evolution and suggest that ongoing surveillance of V. cholerae, ICP1, and PLE in Bangladesh is important for tracking genetic developments relevant to pandemic cholera that can occur over relatively short timescales.

## INTRODUCTION

Pandemics of the severe intestinal disease cholera have plagued humanity on a global scale for over two centuries. Caused by the bacterium Vibrio cholerae, the first pandemic is thought to have occurred in the early to mid-19th century, originating near the Bay of Bengal and sweeping across the Indian subcontinent, Asia, and parts of east Africa (1). There have been six subsequent pandemics, costing millions of lives and culminating in the current 7th cholera pandemic, which accounts for ∼120,000 deaths per year globally (2) and has spread heavily throughout Africa and Asia. Devastating outbreaks have also occurred in the Americas, Europe, Oceania, and the Middle East. The genetic history of the 7th pandemic is marked by several temporally overlapping waves of distinct V. cholerae lineages, which themselves are comprised of numerous inferred transmission events (3). A hallmark of V. cholerae’s evolution is that not only do new lineages frequently arise but also that prior lineages simultaneously disappear. Pandemic V. cholerae is principally the O1 serogroup that is divided into classical and El Tor biotypes (4). A particularly dramatic example of lineage replacement in V. cholerae is that the first six pandemics are thought to have been entirely O1/classical strains, while the 7th pandemic is predominantly O1/EL Tor (5).

Isolates from within the waves of the current pandemic are highly homogenous, and each can be traced back to the Bay of Bengal as the original source (6). Cholera outbreaks are endemic to communities surrounding this area, where it is thought that V. cholerae is in continuous circulation between the human population and estuarian environments that act as the natural reservoir (7, 8). V. cholerae is thought to undergo the majority of its diversification and selection in the aquatic reservoir, giving rise to novel lineages that can infect local communities, spread to cities, and, from there, spread globally (9). Due to this single-point origin of epidemic V. cholerae, surveillance of cholera along the coast of the Bay of Bengal, specifically in Bangladesh, has previously proven very important in identifying not only novel bacterial genotypes (10) but also factors in V. cholerae’s ecosystem that influence the competition between lineages and help shape the selection of genotypes that cause outbreaks (11, 12). Of these factors, bacteriophage predation is likely of particular importance given that phages specific to V. cholerae are found in water sources in regions where the disease is endemic as well as in cholera patient stool (12–14). With phage predation threatening V. cholerae throughout its life cycle, the fitness of epidemic strains is likely contingent on the evolution of phage defense mechanisms.

Of the bacteriophages known to target V. cholerae, ICP1 stands out for its persistence, having first been detected in 1992 in India (15) and consistently recovered in stool samples from Bangladesh since then (14–17). However, V. cholerae can defend against ICP1 through the acquisition of parasitic antiphage islands called PLEs (phage-inducible chromosomal island-like elements) (18). During the course of ICP1 infection, the integrated PLE excises from the chromosome (19), replicates (20), hijacks ICP1 machinery to package its own genome (21), and can accelerate lysis of the host V. cholerae host (22), halting spread of ICP1 to neighboring cells. Five distinct yet highly similar PLEs have been detected in V. cholerae isolates going back to 1949, and over time each variant rose to prominence before being replaced by another (18). This succession is likely reflective of the fact that ICP1 has also evolved counterdefenses against PLEs, including an endonuclease called Odn (23) and, remarkably, a fully functional CRISPR-Cas system (10). However, the presence of these two anti-PLE elements also fluctuates over time, as they target different PLEs and do not cooccur in the same genome (23). The earliest ICP1 isolates from Bangladesh in 2001 possess Odn, while CRISPR-Cas was first was detected in an ICP1 isolate from 2003 (15). Between 2006 and 2017, all ICP1 isolates from Bangladesh were CRISPR(+) (16). However, contemporary coevolutionary trends of ICP1 and PLE are currently unknown.

A recent study from our lab of V. cholerae isolates from cholera patient stool samples in Bangladesh between 2016 and 2019 found evidence of temporal fluctuations in ICP1’s antagonism with other mobile genetic elements and SXT integrative and conjugative elements (24). Considering these findings, we were interested in examining how the dynamics between PLE and ICP1 have progressed during this time period. Surprisingly, we found that after dominating the epidemic landscape for nearly a decade (16), PLE disappeared almost completely in 2018. This disappearance coincided with a switch from CRISPR-Cas back to *odn* among the temporally concurrent ICP1. A phylogenetic comparison of all 148 V. cholerae isolates during the surveillance period revealed an abrupt and nearly total shift from one V. cholerae lineage to a new lineage lacking PLE. Remarkably, this transition occurred over the course of only a few months. We briefly detected a novel PLE, which responds to ICP1 infection and is resistant to the anti-PLE nuclease Odn. An expanded analysis of >3,000 V. cholerae genomes revealed the existence of additional novel PLEs and provides a clearer picture of PLE succession both geographically and temporally. We also compared V. cholerae isolates from within individual patients, revealing heterogenous intrapatient PLE(+)/PLE(−) populations. Similarly, heterogenous ICP1 phages possessing CRISPR-Cas or Odn were isolated from a single patient and exhibited evidence of *in situ* recombination, underscoring the potential for rapid phage evolution in the human gut.

## RESULTS

### Clinical surveillance of Vibrio cholerae and a predatory bacteriophage uncovers regressive shifts in the arms race.

**FIG 1:**
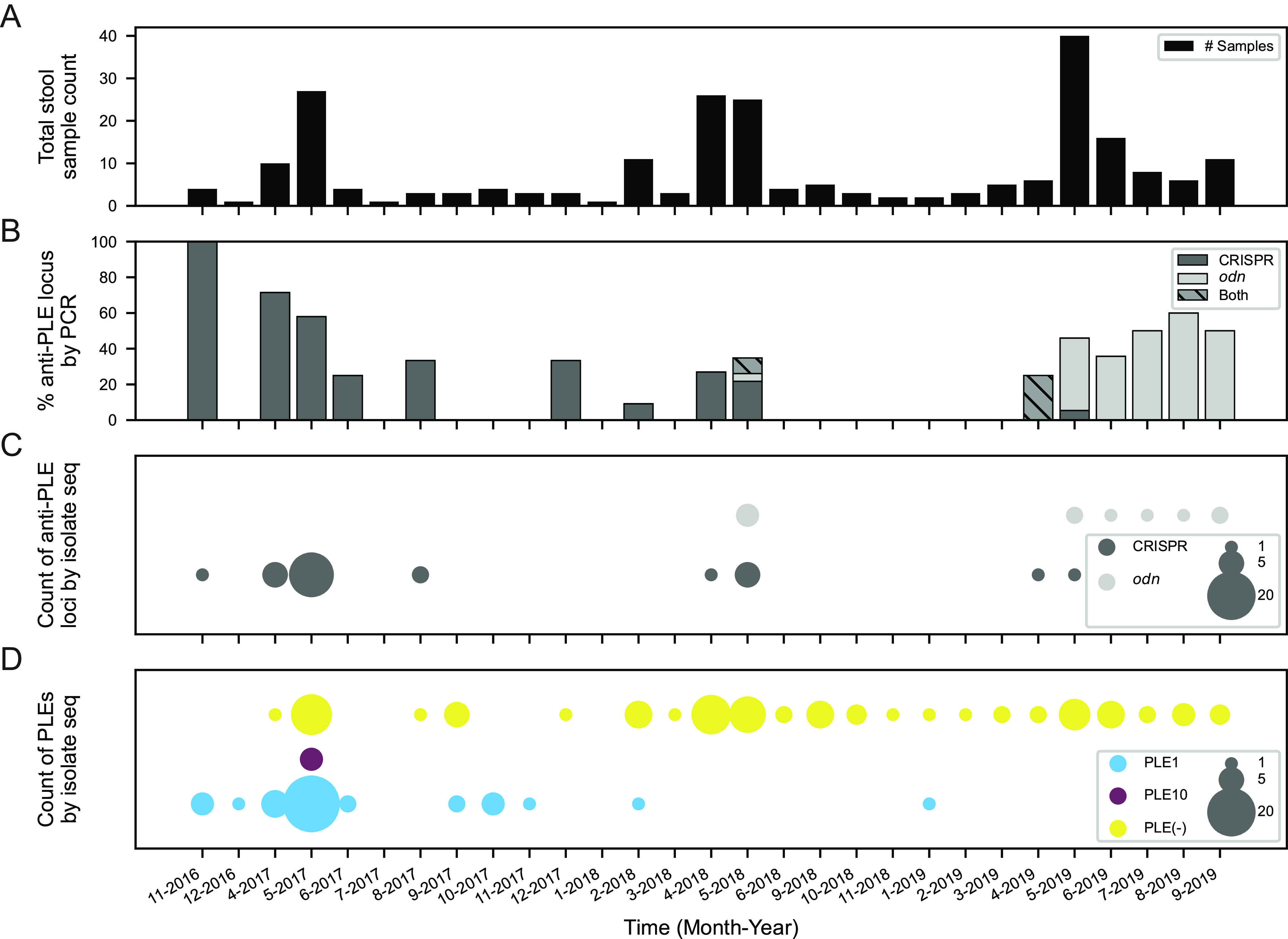
Surveillance of V. cholerae PLE and ICP1 anti-PLE loci in Bangladesh. (A) Total counts of patient stool samples collected and analyzed by month and year of collection (239 total samples) (Data Set S1). (B) Percentage of stool samples in a given month that screened PCR positive for ICP1-encoded anti-PLE counterdefense loci CRISPR-Cas (*n* = 36), *odn* (*n* = 32), or both (*n* = 3). (C) Number of whole-genome-sequenced ICP1 isolates (44 total) from stool samples over the surveillance period by the type of anti-PLE counterdefense locus CRISPR-Cas or *odn*. (D) Number of whole-genome-sequenced V. cholerae isolates (148 total) from stool samples over the surveillance period by PLE variant type. PLE(−) indicates no PLE detected.

We analyzed 239 stool samples from patients in Bangladesh with suspected cholera infections, as determined by a rapid diagnostic test commonly referred to as a dipstick test. These samples were recently interrogated to study the temporal dynamics of SXT integrative and conjugative elements as well as the relevant mechanisms of phage counteradaptation (24). Samples were collected between November 2016 and September 2019, with 119 originating from the capital city Dhaka and 121 from a small city, Mathbaria, located on the Bay of Bengal coast (Fig. 1A; see also Table S1 in the supplemental material).

10.1128/mBio.03088-21.10 TABLE S1 Reagents and resources. Download Table S1, DOCX file, 0.02 MB. Copyright © 2022 Angermeyer et al. 2022 Angermeyer et al. https://creativecommons.org/licenses/by/4.0/ This content is distributed under the terms of the Creative Commons Attribution 4.0 International license.

10.1128/mBio.03088-21.6 DATA SET S1 Metadata for stool samples, V. cholerae, and ICP1 isolates. Download Data Set S1, XLSX file, 0.02 MB. Copyright © 2022 Angermeyer et al. 2022 Angermeyer et al. https://creativecommons.org/licenses/by/4.0/ This content is distributed under the terms of the Creative Commons Attribution 4.0 International license.

Interestingly, ICP1 phages, which previously encoded only CRISPR-Cas in Bangladeshi stool samples after 2006 (16, 17), transitioned back to encoding *odn* around May 2018. Bulk metagenomic genomic DNA was extracted from 197 of the stool samples and used as a template for PCR to detect the presence of ICP1 by screening for *odn* and CRISPR. Notably, all ICP1 isolates sequenced thus far encode either *odn* or CRISPR-Cas, suggesting this PCR screen is a sufficient indicator for the presence of ICP1 in patient samples. This screen revealed that 71 of these 197 samples were positive for ICP1, with 36 (50.7%) samples possessing CRISPR, 32 (45.1%) possessing *odn*, and three (4.2%) having a mix of both anti-PLE loci (Fig. 1B). A sampling period over ∼11 months followed where no ICP1 was detected, and then in April-May of 2019, both loci were detected again. In the final 4 months of surveillance (June to September 2019), only *odn* was detected. To further investigate this transition from CRISPR dominance to apparent *odn* dominance, we examined whole-genome sequences of 44 individual ICP1 isolates from these stool samples (Table S1). These genomes corroborated the PCR results that CRISPR-Cas dominance declined while the *odn* locus began to dominate in mid-2018 (Fig. 1C).

Since both ICP1-encoded loci defend against V. cholerae-encoded PLEs (23), we examined whole-genome sequences of V. cholerae (*n* = 148) from 110 stool samples spanning this period. From the genomes we determined whether each isolate contained PLE and, if so, which of the five known PLEs was present (Fig. 1D). This analysis led to two unexpected results. First, PLE1, which was observed to be the dominant PLE in Bangladesh during previous surveillance (16), appears to have disappeared almost entirely in favor of PLE(−) strains. Interestingly, the disappearance of PLE1 roughly correlated with the period of ICP1’s transition from CRISPR-Cas to *odn*, with only a single isolate possessing PLE1 after February 2018. Second, we observed that four V. cholerae isolates from one stool sample collected in May 2017 possessed a novel PLE variant, which we have designated PLE10. This new PLE was only observed once and was not detected in any later isolates. From these data it is difficult to say whether PLE10 provided inadequate defense against ICP1 and was selected against in the population or if increased surveillance and isolate sequencing would reveal additional instances of PLE10 in other samples.

### PLEs have evolved and diversified both globally and temporally.

The discovery of a novel PLE and the surprising observation that PLEs in Bangladeshi V. cholerae isolates have diminished in recent years prompted us to look more broadly, both geographically and temporally, at other available V. cholerae genomes. Previous surveillance studies tracked the occurrence of PLEs to a limited extent (∼200 genomes) (16, 18); however, this was likely insufficient to fully elucidate the flux of PLEs over time and space. To build on these analyses, we constructed a database of 3,363 sequenced V. cholerae genomes, including our new surveillance isolates and raw reads or fully assembled genomes from public repositories. This collection spans over a century (1916 to 2019) and includes strains collected across 25 different countries (Data Set S2).

10.1128/mBio.03088-21.7 DATA SET S2 Sequenced V. cholerae collection analyzed for PLE. Download Data Set S2, XLSX file, 0.1 MB. Copyright © 2022 Angermeyer et al. 2022 Angermeyer et al. https://creativecommons.org/licenses/by/4.0/ This content is distributed under the terms of the Creative Commons Attribution 4.0 International license.

**FIG 2:**
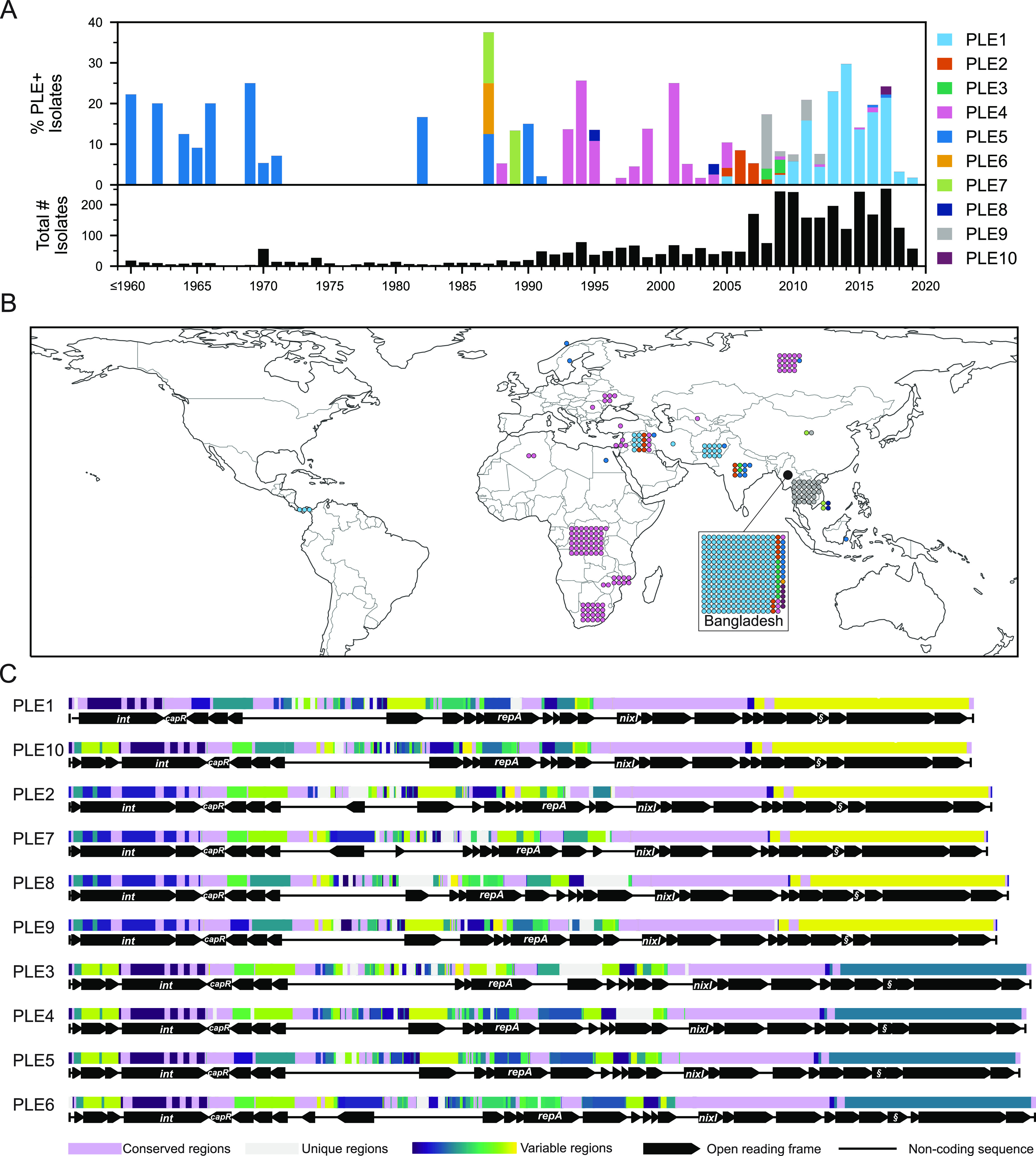
Genetic, temporal, and spatial variability of PLEs in >3,000 V. cholerae genomes collected between 1916 and 2019. (A) Timeline of PLE-positive strains colored by PLE variant type from 3,363 V. cholerae whole-genome sequences. The lower histogram provides the total number of strains per year, while the upper histogram represents a stacked percentage of those that are positive for each PLE. Because of the small number of sequenced genomes from isolates prior to 1960, those genomes were collapsed into a single column. Metadata for all strains analyzed can be found in Data Set S2. (B) Overview of the geographical occurrences of PLE(+) isolates by country of isolation. Each individual isolate is indicated with a colored dot corresponding to the PLE type, as in panel A, and grouped with all others for a given country. The relatively large numbers of isolates from Bangladesh are represented in the inset. (C) Global alignment of all 10 PLEs using progressiveMauve. The order was determined by phylogenetic distance between PLEs (Fig. S1). The mauve-colored regions are conserved across all PLE sequences, while the light gray regions are unique to that specific variant. All other regions vary in conservation between two or more PLEs and were assigned random colors from the variable palette. Annotated genes are shown to scale as black arrows, and several genes with known functions are labeled (§, *lidI*).

Using the previously discovered five PLEs as queries (18), we performed BLASTn searches against each genome and identified four additional PLEs that, along with PLE10, double the total number of known PLE variants from 5 to 10. The abundance of these 10 PLEs has shifted over time, with PLE5 being the only PLE detected before 1987, followed by the other PLEs, culminating in the more recent dominance of PLE1 (Fig. 2A). Previously, PLE5 had been observed as early as 1949, but here we detected PLE5 in an isolate from 1931, increasing the known time range of PLEs circulating in V. cholerae. Generally speaking, the previous pattern of temporal succession, where one PLE dominates for a time before being supplanted by another PLE (16, 18), was observed in this larger collection of isolates, corroborating previous analyses. However, we observed more temporal overlap of PLEs than has previously been appreciated. Furthermore, we observed that while PLE5 had appeared to go extinct among clinical isolates in 1991, three additional PLE5-positive isolates were detected in 2016 to 2017 after a nearly 30-year absence, pointing to unsampled reservoirs in nature where genotypes may persist for some time.

10.1128/mBio.03088-21.1 FIG S1 Global alignment of all 10 PLEs, including phylogeny. The mauve-colored regions are conserved across all PLE sequences, while the light grey regions are unique to that specific variant. All other regions vary in conservation between two or more PLEs and were assigned random colors from the variable palette. Annotated genes are shown to scale as black arrows, and several genes with known functions are labeled (§, *lidI*). The alignment’s final guide tree on the right provides an approximation of phylogenetic distance and determines the order of sequences. The scale represents the number of nucleotide substitutions per base pair. Download FIG S1, EPS file, 2 MB. Copyright © 2022 Angermeyer et al. 2022 Angermeyer et al. https://creativecommons.org/licenses/by/4.0/ This content is distributed under the terms of the Creative Commons Attribution 4.0 International license.

Some of the newly discovered PLE variants, however, only appeared for a short time, such as PLE6, which was detected once in Bangladesh in 1987, and PLE8 in Vietnam in 1995 and 2004. We also detected one possible phage satellite with partial genetic relationship to PLEs, as has been described in non-cholera vibrios recently (25); however, its sequence could not be fully resolved from available data. It is highly likely that we still have not captured the full extent of PLE diversity and variant flux over time, as V. cholerae isolate sampling was sporadic and limited until recent decades (with over half of all isolates collected in only the last 9 years). For example, in this current surveillance effort we observed PLE10 in only a single patient sample, which likely would not have been observed at all with slightly less sampling depth. Whether these short-lived PLEs (and divergent satellites) are somehow less adept at parasitizing ICP1, are selected against by specific anti-PLE counterdefenses, or are victims of simple stochastic fluctuations is unknown.

Additionally, transmission bottlenecks that likely occur in the course of regional outbreaks may constrain PLEs geographically (Fig. 2B), such as in the two most recent outbreaks in Haiti (26) and Yemen (27), where no PLEs were detected. The most notable exception is Bangladesh, where 7 of the 10 PLE variants have been detected. While this may be partially due to the sampling focus on this region, as a significant fraction (∼20%) of the V. cholerae genomes analyzed were from there, it also may be due to its juxtaposition with the Bay of Bengal, where the majority of V. cholerae’s diversification and evolution are predicted to occur.

Having found additional PLEs, we then performed a whole-genome alignment of all 10 PLE variants (Fig. 2C), which was used to generate a phylogenetic tree (Fig. S1). All PLEs are broadly syntenic and share numerous regions of nucleotide conservation. Annotation and sequence comparison of open reading frames confirmed that the newly detected PLEs 6 to 10 possess genes key to PLE function that have been characterized in the previously studied PLEs 1 to 5. These include *nixI*, which encodes a nickase that interferes with ICP1’s ability to replicate (25), *lidI*, which can accelerate lysis of the V. cholerae host cell (22), and *capR*, which acts to repress ICP1’s capsid morphogenesis operon (21). The conservation of these genes indicates that the newly identified PLEs play roles similar to those of PLEs 1 to 5 as parasites of ICP1. Two additional genes indicate that these PLEs are also still functional as mobilizable genetic elements: an integrase that catalyzes PLE excision from the host genome and integration following transduction, and a replication initiation factor that controls PLE replication.

PLEs encode one of two integrases (Fig. S2), the PLE1-type (found in PLEs 1, 3, 4, 5, 6, and 10), which directs integration into a Vibrio cholerae repeat (VCR) sequence in the superintegron and recognizes ICP1-encoded PexA as a recombination directionality factor to catalyze PLE excision (19), and the PLE2-type (found in PLEs 2, 7, 8, and 9), which directs PLE integration into the M48 family metallopeptidase gene (*vca0581*) and does not interact with PexA. VCRs are ∼124-bp repeat elements that are interspersed between gene cassettes within the superintegron (28). VCRs are extremely conserved, which could allow PLEs with the PLE1-type integrase to integrate into any single VCR following horizontal transfer, resulting in isogenic PLEs flanked by different genes in the superintegron. Previous analyses showed that PLE1 integrates into various distinct VCRs under laboratory conditions, but such variability has not been observed among PLE(+) V. cholerae isolates from clinical specimens, suggesting that PLE transmission is largely vertical in nature (16, 18). To determine if this finding held true, we extracted the genomic regions flanking PLE for every PLE-positive V. cholerae isolate in our data set. While no flanking differences were observed among the PLEs with PLE2-type integrases, we identified 14 unique VCR integration sites for the PLE1-type integrase-positive V. cholerae isolates in our database (Fig. S3 and Data Set S3). While most PLE variants appeared to have a single integration site, either through preferential horizontal integration or vertical transmission, we did detect multiple sites for PLE1, PLE4, and PLE5, indicating horizontal acquisition events in nature.

10.1128/mBio.03088-21.2 FIG S2 Alignment of PLE-encoded integrases. Coloring is determined by average BLOSUM62 score of pairs of letters in each column: light blue, ≥1; light gray, ≥0.2; other, no color. (A) Alignment of integrase protein from all PLEs. (B) Alignment of only PLE1-type integrases. (C) Alignment of only PLE2-type integrases. Download FIG S2, JPG file, 2 MB. Copyright © 2022 Angermeyer et al. 2022 Angermeyer et al. https://creativecommons.org/licenses/by/4.0/ This content is distributed under the terms of the Creative Commons Attribution 4.0 International license.

10.1128/mBio.03088-21.3 FIG S3 PLE integration sites. PLE abundance at highest-confidence integration sites based on bioinformatically determined flanking regions. Sites 1 to 14 are within VCRs in V. cholerae’s superintegron, while site 15 is in gene *vca0581*. Points are colored by PLE variant (left). Abundance is given by point size and number; unlabeled points are *n* = 1. Detailed information on flanking regions and additional lower-confidence possible integration sites are in Data Set S3. Download FIG S3, EPS file, 1.5 MB. Copyright © 2022 Angermeyer et al. 2022 Angermeyer et al. https://creativecommons.org/licenses/by/4.0/ This content is distributed under the terms of the Creative Commons Attribution 4.0 International license.

10.1128/mBio.03088-21.8 DATA SET S3 PLE integration site chromosomal flanking regions. Download Data Set S3, XLSX file, 0.02 MB. Copyright © 2022 Angermeyer et al. 2022 Angermeyer et al. https://creativecommons.org/licenses/by/4.0/ This content is distributed under the terms of the Creative Commons Attribution 4.0 International license.

The PLE-encoded replication initiation protein RepA has a conserved C terminus across all PLEs but varies at the N terminus, which directs DNA binding to the PLE origin of replication (*ori*) (20). Previous work found that RepA’s N terminus for PLEs 1 to 5 fell into two groups, one with PLEs 1, 4, and 5 and the second with PLEs 2 and 3. Diversification of the PLE replication module (comprised of a compatible RepA and *ori*) is hypothesized to be driven by ICP1’s nuclease Odn, which mimics the PLE1,4,5-type RepA protein to bind to the cognate *ori* and then cleave it. For PLE to escape Odn by modifying its *ori*, it must possess a compatible RepA N terminus as well as the conserved C terminus, which is hypothesized to facilitate recruitment of the replisome machinery from ICP1 (20). Including the five new PLEs described here, phylogenetic analysis revealed the existence of a novel third RepA N-terminal variant found in PLEs 6, 8, 9, and 10 (Fig. S4). In support of the designation of a third type of PLE replication module, previously validated *ori* sequences in PLE1-5 were not observed in PLEs with the PLE10-type RepA protein, indicating that these PLEs harbor a compatible unique *ori* sequence that would be resistant to Odn-mediated cleavage.

10.1128/mBio.03088-21.4 FIG S4 RepA alignment and phylogenetic distance. (Left) Alignment of PLE-encoded RepAs where coloring is determined by average BLOSUM62 score of pairs of letters in each column: light blue, ≥1; light gray, ≥0.2; other, no color. (Right) Phylogenetic tree of RepA amino acid alignment with major groups highlighted. Download FIG S4, JPG file, 1 MB. Copyright © 2022 Angermeyer et al. 2022 Angermeyer et al. https://creativecommons.org/licenses/by/4.0/ This content is distributed under the terms of the Creative Commons Attribution 4.0 International license.

### PLE10 responds to ICP1 infection and is resistant to Odn.

**FIG 3:**
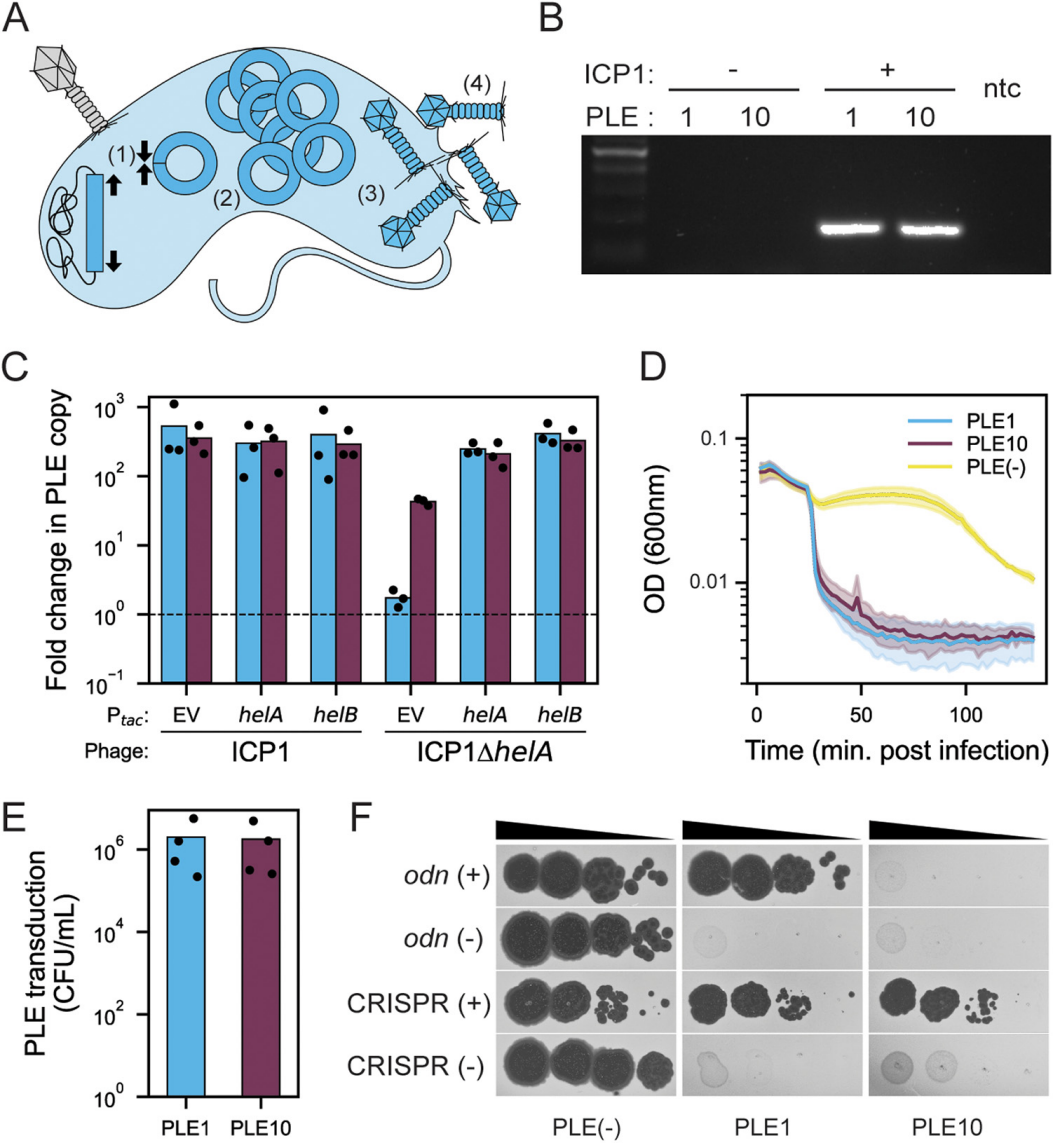
PLE10 exhibits excision, replication, and packaging in response to ICP1 infection and evades the anti-PLE nuclease Odn. (A) Model showing the steps of the PLE-ICP1 interaction in a V. cholerae host cell. (1) The integrated PLE (in dark blue) excises and circularizes upon ICP1 infection. (2) PLE replicates and (3) is packaged into modified phage particles. (4) PLE transducing-particles are released through cell lysis, which occurs on an accelerated timeline. (B) Agarose gel of PCR products to detect circularized PLE in uninfected V. cholerae and following infection by ICP1^2006^ lacking CRISPR-Cas (the phage isolate previously used to probe PLE circularization [19]). The lane on the far left is the ladder, and that on the far right is the no template control (ntc). (C) Quantification of change in PLE1 (blue) and PLE10 (purple) copy number 30 min after infection with ICP1^2006^ lacking CRISPR-Cas or the Δ*helA* mutant (the phage isolate previously used to probe PLE replication [18, 20, 29]). IPTG-inducible plasmid constructs (P*_tac_*) were induced prior to phage infection. EV is the empty vector control. The dashed line indicates no change in copy number compared to the sample taken prior to phage infection (see Materials and Methods). (D) Lysis curves of V. cholerae harboring PLE1 or PLE10 following infection by ICP1^2006^ lacking CRISPR-Cas (the phage isolate previously used to probe lysis kinetics [18, 22]) versus the PLE(−) control. (E) PLE-transducing particles generated during infection with ICP1^2006^ lacking CRISPR-Cas (the phage isolate previously used to probe PLE transduction [18]). (F) Tenfold dilutions of the phage isolate carrying the anti-PLE locus indicated or mutant derivative spotted on V. cholerae with the PLE indicated (bacterial lawns are in gray, zones of killing are in black). Contemporary ICP1 isolates were used in this assay: the CRISPR(+) isolate with spacers against PLE1 and PLE10, ICP1^2017^, was recovered from the same patient sample as the original PLE10(+) V. cholerae, and an Odn(+) isolate, ICP1^2019^, were used. See Table S1 for a complete description of phage isolates. For experiments in panels B to F, PLEs were transduced into the same genetic background (V. cholerae E7946); see Table S1 for strain details.

All previously studied PLEs (PLEs 1 to 5) are triggered by ICP1 infection to excise, replicate, and package themselves into transducing particles using ICP1’s machinery (Fig. 3A) (18). Based on genetic similarity to PLEs 1 to 5, we hypothesize that the five new PLEs described here respond similarly to ICP1 infection. Unfortunately, of the new PLE variants we only had access to V. cholerae strains possessing PLE10 and therefore experimentally assessed how this variant responds to ICP1 infection.

To determine if this novel PLE10 variant indeed functions in a way similar to that of known PLEs, we performed several assays in tandem with PLE1. First, a circularization PCR showed that PLE10 excises and circularizes during ICP1 infection (Fig. 3B), as was expected due to the similarity of the PLE1 and PLE10 integrases (Fig. S2). Second, we observed that PLE10 replicates in response to ICP1 infection (Fig. 3C). This confirms that the divergent PLE10 replication module is functional and likely exploits ICP1 machinery to promote PLE replication. Interestingly, although PLEs 1 to 5 do not replicate during infection by ICP1 mutants lacking an accessory SF1B-type helicase (*helA* or *helB*) (29), PLE10 does replicate to low levels in the absence of this ICP1-encoded helicase (Fig. 3C), suggesting that the divergent replication module allows for additional flexibility in PLE’s reliance on ICP1’s replication machinery. Third, we observed that PLE10, which possesses a *lidI* homologue (22), also exhibited an accelerated lysis phenotype, like PLE1, following ICP1 infection (Fig. 3D). Fourth, PLE10 mobilized and transduced its genome to naive V. cholerae hosts during infection at rates similar to those of PLE1 (Fig. 3E). These results demonstrate that PLE10 exhibits the anticipated response to ICP1 infection, namely, excision, replication, and transduction.

As ICP1 is known to possess two anti-PLE counterdefense loci (*odn* and CRISPR-Cas), we tested the ability of ICP1 with either loci to plaque on V. cholerae having either PLE1 or PLE10. PLE1 is susceptible to ICP1 possessing CRISPR-Cas (those with a spacer specific to PLE1) (10), and one hypothesis is that this new PLE10 variant has an advantage over PLE1 through the acquisition of an anti-CRISPR, although none have been found previously in PLEs. However, we found that the CRISPR-Cas(+) phage, isolated from the same stool sample as the PLE10 V. cholerae isolates and naturally possessing spacers specific to both PLE1 and PLE10, could overcome PLE10 during infection and that this activity was dependent on ICP1’s CRISPR-Cas system (Fig. 3F). This was an identical result to that for the PLE1 control, which was overcome by the same CRISPR-Cas(+) phage. The inability to block plaque formation by CRISPR-Cas(+) phage indicates that PLE10 has no anti-CRISPR protein that might provide an evolutionary advantage over PLE1.

In contrast, an ICP1 phage with *odn* was unable to form plaques on a PLE10(+) host but was able to form plaques on an otherwise isogenic host harboring PLE1 (Fig. 3F). Purified Odn also did not exhibit nucleolytic activity against a probe amplified from the ORF-less region (expected to harbor the *ori*) of PLE10 compared to a probe from PLE1 that was cleaved as expected (Fig. S5). Collectively these data are consistent with the specificity of the origin-targeting behavior of Odn (23) and the divergent PLE10 replication module conferring protection from Odn-mediated cleavage. The *odn*(+) phage, tested for its capacity to form plaques on PLE10, was from a more recent 2019 stool sample, a time period in which *odn*-positive ICP1 phages were dominant in our surveillance (Fig. 1C). However, despite the observation that PLE10 is resistant to this *odn*(+) phage, it did not have an apparent selective advantage over PLE1 during this surveillance period (Fig. 1D).

10.1128/mBio.03088-21.5 FIG S5 Anti-PLE ICP1-encoded origin-directed nuclease (Odn) does not show activity against PLE10. Nuclease assay showing the integrity of a PCR product amplified from the noncoding region containing the *ori* from the PLE variant indicated (numbers) treated with (+) and without (–) 500 nM purified Odn. Shown is a representative of nuclease assays performed in triplicate. Download FIG S5, EPS file, 1.9 MB. Copyright © 2022 Angermeyer et al. 2022 Angermeyer et al. https://creativecommons.org/licenses/by/4.0/ This content is distributed under the terms of the Creative Commons Attribution 4.0 International license.

### Colony and plaque screens reveal intrapatient heterogeneity.

**FIG 4:**
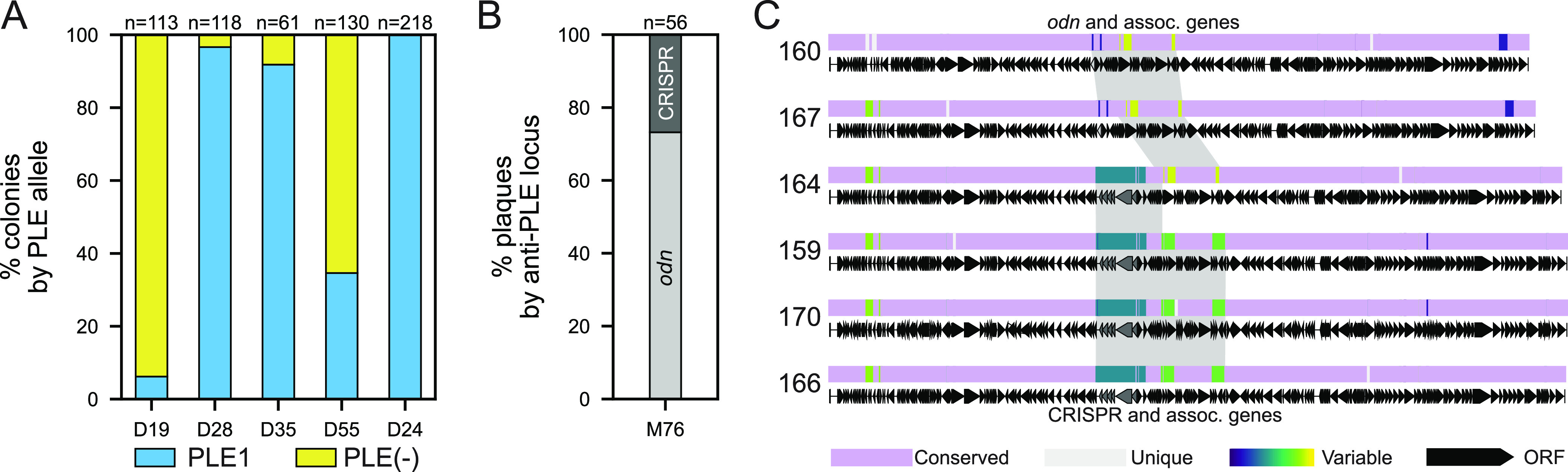
PLE(+)/PLE(−) V. cholerae and ICP1 anti-PLE loci heterogeneity within patients. (A) The fraction of total colonies with and without PLE for V. cholerae isolates within individual stool samples as determined by PCR. The number of colonies screened from each patient sample (given an arbitrary number by date of isolation with the prefix D are from icddr,b in Dhaka) are given above each bar. (B) The fraction of ICP1 plaques with the anti-PLE locus indicated recovered from one stool sample from Mathbaria (M76) as determined by PCR. (C) Alignment of six ICP1 isolates from stool sample M76 (panel B) using progressiveMauve. The mauve-colored regions are conserved across all ICP1 sequences, while the light gray regions are unique to that specific isolate. All other regions vary in conservation between two or more isolates and were assigned random colors from the variable palette. Annotated genes are shown as black arrows, and Cas and *odn* genes are shown in gray. The gray shading highlights regions with *odn* with associated genes (in phages 160 and 167) and CRISPR-Cas with associated genes (in phages 159, 170, and 166) at ≥95% sequence identity. Isolate 164 demonstrates recombination between the CRISPR-Cas region and the *odn*-associated region. See Table S1 for a complete description of phage isolates.

Preliminary PCR screening of metagenomic DNA from stool samples revealed some samples with a positive signal for PLE but from which we later recovered V. cholerae isolates that were PLE(−). This finding prompted us to interrogate several samples further to better understand the ratios of PLE(+) to PLE(−) strains within intrapatient populations. We selected three samples from May 2017 (D19, D28, and D35) and one from September 2017 (D55), as both time points also possessed a mix of PLE(+) and PLE(−) isolates between patient samples (Fig. 1D). We performed PCR on a large number of colonies (*n* = 61 to 130) picked from each of these samples and observed that all samples were indeed heterogenous for the presence of PLE [ranging from 6 to 97% PLE(+)] (Fig. 4A). We additionally chose one sample from May 2017 from which all (*n* = 4) sequenced isolates were PLE(+) to see if PLE heterogeneity could be caused by stochastic loss under laboratory conditions. PCR of 218 colonies revealed all to be PLE(+) (Fig. 4A), indicating that PLE loss was highly unlikely to occur as a result of the outgrowth and isolation procedures used. These results reinforce previous discoveries that V. cholerae can diversify to some extent during human infection (30), although whether this finding indicates a loss or gain of PLE is unclear.

Similarly, we performed PCR on a large number of ICP1 plaques from sample M76 (May 2018), in which the initial screen of metagenomic DNA extracted from stool revealed a mixed signal of both ICP1-encoded *odn* and CRISPR-Cas anti-PLE loci. This was also the first instance of *odn* reappearance (Fig. 1B) in this surveillance period. All previously discovered ICP1 isolates have either *odn* or CRISPR-Cas but never both (15, 17, 23), and these counterdefense mechanisms have always been in the same location within the genome. It is an intriguing possibility that some rare isolates could carry both *odn* and CRISPR-Cas, but PCR results from purified plaques (*n* = 56) from this sample showed that members of this intrapatient ICP1 population indeed only had CRISPR-Cas or *odn* (Fig. 4B).

In addition to the 56 screened plaques, we also sequenced the genomes of six isolates of ICP1 from this patient sample, two *odn*(+) isolates and four CRISPR(+) isolates. Whole-genome sequencing revealed that both *odn*(+) phage had a canonical downstream region typically associated with *odn*, and three of the CRISPR(+) phage had the expected distinct conserved set of associated genes. However, one isolate (2018_Mat_164) harboring the CRISPR-Cas anti-PLE locus possessed the downstream region typically associated with *odn* (Fig. 4C). This appears to be an example of within-patient recombination between two ICP1 genotypes and is strong additional evidence that coinfection is a driver of ICP1 evolution, as has been demonstrated previously under laboratory conditions (22). To the best of our knowledge, this is the first attempt to document intrapatient diversity of vibriophages and, as such, the rates of *in situ* recombination are unknown. Nonetheless, these data highlight that ICP1 evolution occurs in the human gut, giving rise to distinct ICP1 genotypes with distinct host ranges (23) that could differentially impact the fitness of cocirculating V. cholerae.

### V. cholerae isolates from 2016 to 2019 in Bangladesh sort into two distinct phylogenetic lineages.

**FIG 5:**
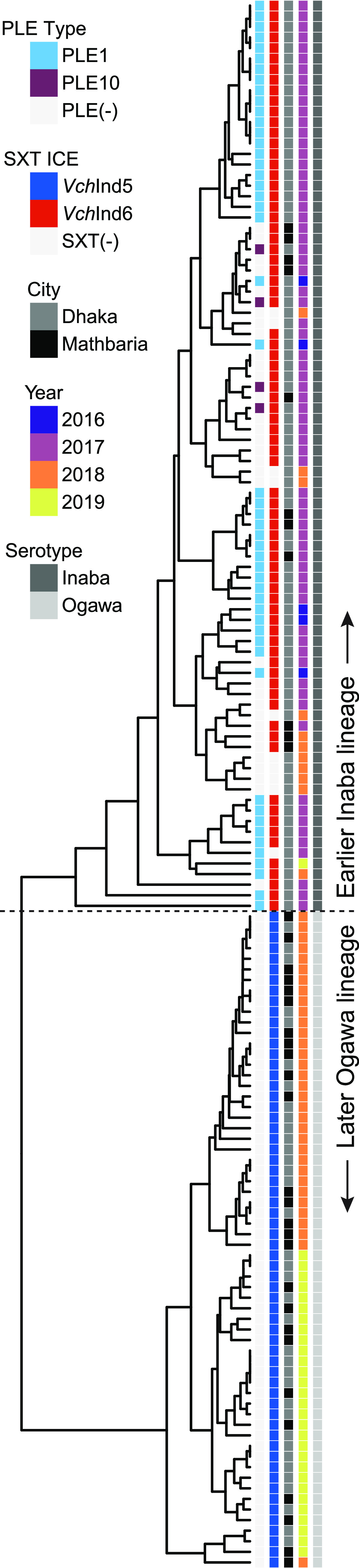
Phylogeny of V. cholerae isolates during the surveillance period in Bangladesh reveals the replacement of a previously prevalent PLE(+) lineage with a PLE(−) lineage in 2018 to 2019. A cladogram of 148 V. cholerae isolates based on 532 variable nucleotide genomic positions (Data Set S4). Columns next to the tree contain metadata for (left to right) the PLE variant present, the SXT ICE present, city of isolation, year isolated, and serotype (based on whole-genome sequencing and analysis of *wbeT*). Column coloring and order is shown in the legend from top to bottom. Strains lacking MGEs (PLE or SXT ICE) are indicated in light gray.

The transmission and evolution of V. cholerae is characterized by successive waves of clonally related strains that are replaced over time (3), and the phylogenetic relationships between these waves generally have been determined by differences in single point mutations. We observed that PLE disappeared from V. cholerae isolates during the course of our surveillance (Fig. 1D); however, we did not know if this is the result of PLE being lost from a clonal population or if the PLE(+) lineage was replaced by a distinct PLE(−) lineage. To address this question, we identified 532 sites with single nucleotide variations (SNVs) (Data Set S4) common across all 148 isolates and used this information to construct a core genome phylogenetic tree (Fig. 5).

10.1128/mBio.03088-21.9 DATA SET S4 SNVs among Bangladesh V. cholerae isolates. Download Data Set S4, XLSX file, 0.2 MB. Copyright © 2022 Angermeyer et al. 2022 Angermeyer et al. https://creativecommons.org/licenses/by/4.0/ This content is distributed under the terms of the Creative Commons Attribution 4.0 International license.

It was immediately clear that the V. cholerae isolates cluster into two highly distinct phylogenetic groups that largely coincide with the presence of PLE, suggesting that we captured a replacement event where the PLE(−) lineage emerged to dominate as opposed to witnessing the loss of this mobile genetic element (MGE) in an otherwise persistent lineage. This was further reinforced by a previous observation in this surveillance data set that another MGE that plays a role in phage defense, integrative and conjugative elements (ICEs) of the SXT/R391 family, exhibited a transition from a mix of *Vch*Ind6 and SXT(−) to *Vch*Ind5 over the same time period (24). Additionally, sequences of the *wbeT* gene, which is part of the biosynthetic pathway for the O1 antigen, also revealed a switch from the Inaba (nonfunctional *wbeT*) to Ogawa (functional *wbeT*) serotype that perfectly tracked with the lineage replacement. In the earlier Inaba lineage, *wbeT* was disrupted by a transposon with an identical sequence and integration site across all isolates. Remarkably, this replacement sweep appears to have occurred over months instead of years, with only a few isolates from the earlier PLE(+)/*Vch*Ind6(+)/Inaba lineage persisting into 2018 and only a single one in 2019. This indicates that the later PLE(−)/*Vch*Ind5(+)/Ogawa lineage has some competitive advantage over the earlier lineage driving its sudden and dominating emergence. We also saw no patterns separating the sampling locations of Dhaka and Mathbaria, indicating that the replacement sweep occurred rapidly geographically as well as temporally.

Finally, in addition to the samples examined by colony PCR (Fig. 4A), multiple isolates were sequenced from several other samples when recoverability permitted. While significantly more isolate sequencing would be required to draw meaningful conclusions about the full extent of intrapatient diversity, we did observe both SNV and MGE variability between isolates in some patient samples (Data Sets S1 and S4). However, in no sample did we detect isolates from both lineages among the intrapatient isolates, perhaps suggesting that although rapid, lineage sweeps are unlikely to occur in the context of a single patient’s infection.

## DISCUSSION

The trajectories of global cholera outbreaks are generally defined by successive waves of V. cholerae in which a new lineage replaces the previous lineage (3). While it is unclear exactly what allows successive lineages to outcompete their predecessors, it is broadly theorized that each new lineage in the current 7th pandemic originated from the areas surrounding the Bay of Bengal (6). Therefore, developing a better understanding of the genetic variability of V. cholerae in this region is likely key to unlocking the mystery surrounding the cycle of global selective sweeps. Furthermore, predatory bacteriophages like ICP1 and antiphage defenses such as PLE may play an important role in determining which lineages become pathogenically relevant in human populations. In this study, we describe findings from continued surveillance of V. cholerae in Bangladesh and build upon previous work to further reinforce the importance of monitoring these microbial populations in communities proximal to the Bay of Bengal.

The antagonism between ICP1 and PLE results in an ongoing arms race in which ICP1 evolves means to target PLE and inhibit PLE’s anti-ICP1 activity. The earliest known ICP1 isolates have the Odn nuclease, which cleaves PLE’s origin of replication (23), while PLEs likely diversified to escape Odn targeting. It was assumed, however, that the extreme flexibility and rapid adaptation provided by the later acquisition of a novel CRISPR-Cas system had given ICP1 the upper hand in this battle (16), or at least that CRISPR-Cas would be so beneficial that it would remain fixed in the ICP1 population. Remarkably, though, we observed that despite the assumed superiority of a CRISPR-Cas counterdefense mechanism, a reversion occurred in the ICP1 population back to carrying the *odn* locus instead of CRISPR-Cas. During this same time frame before reversion to *odn*, we also found that nearly all V. cholerae isolates lacked PLE. Whether or not these two nearly simultaneous changes are functionally connected is unclear. Perhaps the much larger CRISPR-Cas locus is too burdensome for the phage to encode when PLE no longer threatens its viability, maybe *odn* is more effective at fully restricting PLE activity for the PLEs that it can target, or possibly both (23). These hypotheses suggest that PLE was lost from the V. cholerae population first, and in the absence of that selective pressure, ICP1 quickly reverted to a more favorable genotype.

A similar reversion event was previously observed in these same surveillance samples in which the promoter for *orbA* that inhibits antiphage components of the SXT ICE *Vch*Ind5 was missing from ICP1 isolates while *Vch*Ind5 was absent from the V. cholerae population and a different SXT ICE dominated. However, when *Vch*Ind5 became dominant in the population around mid-2018 (Fig. 5), phage isolates with *orbA* and its functional wild-type promoter also reemerged as the dominant ICP1 genotype (24). It is possible that ICP1’s robust accessory genome allows it to keep pace with emergent V. cholerae lineages, and reemergence of historical genotypes suggests unsampled reservoirs of ICP1 diversity, much like we expect for V. cholerae. If future lineage shifts cause PLE to reemerge in the V. cholerae population, it may also mean that CRISPR-Cas, or perhaps yet another anti-PLE mechanism, will again supplant Odn in the ICP1 population. However, this hypothesis is potentially weakened by the discovery of a new PLE in one patient stool sample approximately a year before the shift in ICP1’s anti-PLE loci. The new PLE (PLE10) was shown to be functional as a phage parasite in response to ICP1 infection but was also resistant to Odn targeting because it harbors a novel origin of replication. In this case, why did PLE10 not proliferate in the V. cholerae population after ICP1 had switched to *odn*? It is impossible to say for certain, but it may have some unknown competitive disadvantage or was simply unable to spread through the population while CRISPR-Cas with PLE10 spacers was still cooccurring. It is further possible that the pressures inherent in the dynamics of ICP1-PLE interactions are not responsible for driving V. cholerae lineage selection and PLE10 was simply not present in the lineage that swept to dominance in 2018.

In further investigating the genomics of this lineage switch among the V. cholerae surveillance isolates, we observed two very distinct clades based on SNVs. While the SNV analysis did not reveal any candidate mutations that would indicate an obvious competitive advantage of the new lineage, a sizable number of the nonsynonymous variations were perfectly separated between the two lineages (see Data Set S4 in the supplemental material). This indicates that the lineage shift reflects an emergence of a distinct V. cholerae variant that already had these mutations in its genome. Additionally, the serotype switch from Inaba to Ogawa also tracked precisely with the two lineages. The tendency of V. cholerae to change serotype between Inaba and Ogawa, or vice versa, has been well documented. This is in large part due to the ease with which serotype agglutination tests can be performed and has revealed these shifts to be very common (31). This phenomenon has been documented globally (32, 33) as well as in Bangladesh for almost five decades (34), including a time range that overlaps in part with our surveillance period and agrees with the observation made here (35). Despite this long history, it is still unknown what drives the Inaba/Ogawa serotype switching in V. cholerae. As the function of the *wbeT* gene is to modify the O1 anitgen, it has been hypothesized that phage predation is a selective pressure (36); however, ICP1 (which uses the O1 antigen as its receptor) is insensitive to this modification (14), and there is no evidence to indicate that the other two known vibriophages frequently isolated from patient stool samples (ICP2 and ICP3) are impacted by such a modification. An alternative hypothesis is that O1 antibodies created as an immune response to V. cholerae in patient populations could select against whichever serogroup is currently circulating. While this has not been conclusively tested, a recent study found that patients challenged with an Inaba strain developed antibodies that cross-reacted effectively with the Ogawa serotype (37), suggesting a limited selective advantage to serotype switching in evading human immunity.

We did find some evidence of possible diversification within patients by detecting mixed populations of PLE(+) and PLE(−) colonies from several patient samples as well as in sequenced isolates from one patient that varied by several SNVs. These observations are reinforced by previous discoveries that found genetic variation of V. cholerae during human infection (30) and even the emergence of hypermutator phenotypes (38). Whether this indicates an *in situ* loss, possibly due to an incurred fitness cost of harboring the MGE, gain of PLE through lateral transfer, or coinfection with distinct strains is unknown. We also found evidence of bacteriophage heterogeneity leading to recombination within a single patient. We are not aware of this being observed in nature before, and there is experimental evidence of ICP1 recombination under laboratory conditions (22). The evolutionary variation in the V. cholerae/ICP1/PLE system is likely apparent, and perhaps more so, in the aquatic reservoir. A deeper investigation of individual patient samples as well as longitudinal surveillance and environmental samples will be important to begin to fully understand the dynamics at play in driving V. cholerae evolution and lineage selection.

## MATERIALS AND METHODS

### Bacterial growth conditions.

The bacterial strains and plasmids used in this study are listed in Table S1 in the supplemental material. All bacterial strains were grown at 37°C in LB (Fisher) with aeration or on LB agar plates. Antibiotics were used when appropriate (100 μg/mL streptomycin, 75 μg/mL kanamycin; 2.5 μg/mL chloramphenicol for V. cholerae and 25 μg/mL for E. coli). Ectopic expression constructs in V. cholerae were induced 20 min prior to ICP1 infection with 1 mM isopropyl β-d-1-thiogalactopyranoside (IPTG) and 1.5 mM theophylline.

### Phage growth conditions.

The phages in this study are listed in Table S1. Bacteriophages were propagated on V. cholerae, harvested via polyethylene glycol precipitation (39) or medium exchange on Millipore’s Amicon Ultra centrifugal filters (40), and quantified via the soft-agar overlay method (39).

### Isolation of bacteria, phage, and total DNA from stool.

The collection of stool samples and isolation of V. cholerae and phages interrogated in this study was recently described (24). Briefly, deidentified rice water stool (RWS) from patients presenting with diarrheal disease were tested for the presence of V. cholerae via a crystal V. cholerae rapid dipstick test (RDT; Span Diagnostics, Surat, India) at icddr,b Dhaka Hospital and Government Health Complex of Mathbaria, Pirojpur, Bangladesh. RDT-positive stool samples were mixed with glycerol and frozen for transport to the University of California, Berkeley. V. cholerae was isolated from RDT-positive stool samples onsite in Bangladesh following enrichment in alkaline peptone water (APW) and plating on taurocholate tellurite gelatin agar (TTGA) (Difco). Additional isolates of V. cholerae from these RWS samples were obtained following APW outgrowth and culturing thiosulfate-citrate-bile salts-sucrose agar (Fisher) and Vibrio ChromoSelect agar (Sigma) at the University of California, Berkeley. To isolate phages, dilutions of RWS and APW outgrowths were mixed with log-phase V. cholerae and plated in 0.3 to 0.5% LB top agar. Total DNA was extracted from stool samples using the DNeasy PowerSoil kit (Qiagen) with 333 μL of stool by following a modified extraction protocol. Briefly, 200 μL of bead solution was removed and replaced with 200 μL of phenol-chloroform-isoamyl alcohol (pH 7 to 8; Sigma) before the stool was added. High liquid content of stool resulted in increased sample volume after bead beating, resulting in our scaling up of reagent volumes, after which the manufacturer’s instructions were followed.

### Whole-genome sequencing.

Whole-genome sequencing of V. cholerae and ICP1 isolates during the surveillance period was performed in our previous study (24). Briefly, DNA from V. cholerae and phages was extracted using commercially available kits (Qiagen DNeasy blood and tissue or monarch genomic DNA purification kit from New England BioLabs). For preparation of phage DNA, phage stocks were treated with DNase at 37°C for 30 min before heat inactivation prior to DNA extraction by following the manufacturer’s instructions. Libraries for Illumina sequencing were prepared with New England BioLabs’s Ultra II DNA or Ultra II FS DNA prep kits or performed by the Microbial Genome Sequencing Center. Sequencing (150- by 150-bp paired end) was performed by the QB3 Genomics Core at the University of California, Berkeley, or by the Microbial Genome Sequencing Center.

### CRISPR/Odn and PLE PCR screening.

To detect ICP1-encoded anti-PLE loci from patient stool, total DNA from stool was used as a template for PCR using the primers listed in Table S1. Colony PCR using the primers listed in Table S1 was performed on individual V. cholerae colonies to screen for the presence of PLE1 among isolates recovered from the same patient sample.

### Genome database and PLE discovery.

A database of publicly available genomes was generated by downloading all deduplicated assemblies from the NCBI V. cholerae genome directory (https://www.ncbi.nlm.nih.gov/genome/browse/#!/prokaryotes/505/) and by a literature search for raw sequence reads available from the NCBI Sequence Read Archive (https://www.ncbi.nlm.nih.gov/sra). These raw reads were subsequently downloaded in fastq format and assembled with spades v3.14.0 using default settings. This data set is similar to but expanded from that of a previous work (24). Each of the five previously known PLEs were used as BLASTn queries against the completed V. cholerae database, and the resulting nonoverlapping hit lengths (E value of ≤0.001) were summed for each PLE. Summed hit lengths that exceeded 10% of that PLE’s total nucleotide length were considered putative PLEs and were manually curated with CLC Main Workbench 7 to ensure similar length, synteny, and sequence similarity to known PLEs. This BLASTn approach was also used to identify genomes with copies of the known PLEs.

### PLE alignment and phylogeny.

PLE fasta files were aligned with progressiveMauve (v. 2015-02-25) using default parameters as well as the assume colinear genomes setting. Region color combinations were randomly chosen from the Viridis color palette for each combination of PLEs, and the phylogenetic tree was generated from the guide_tree file.

### PLE integration sites.

The first 100 bp of the genomic regions immediately flanking every PLE-positive V. cholerae isolate in the V. cholerae genome database were extracted and grouped by those that matched exactly. Only PLEs with a uniquely matched set of left and right regions flanking the integration site were considered (i.e., no left flank sequence occurred with more than one right flank sequence). This ensured that integration site differences were not due to genomic variability or shuffling within the superintegron of each PLE-positive V. cholerae isolate. Those PLEs that satisfied these requirements were grouped by identical flank pairs (i.e., integration sites) and plotted.

### Generation of bacterial and phage mutants.

PCR constructs for bacterial mutations of interest were made through splicing by overlap extension and introduced by natural transformation (41). To generate a PLE10(+) strain in the V. cholerae E7946 background, PLE transduction was performed with magnesium as previously described (21). Phage mutants were constructed using CRISPR-Cas engineering (42). All mutant strains were verified with Sanger sequencing over the region of interest.

### Assays for PLE’s response to ICP1 infection.

Circularization of PLE upon infection was completed as previously described (19), with slight modifications. Briefly, V. cholerae strains were grown to an optical density at 600 nm (OD_600_) of ∼0.3, at which point an uninfected sample was taken, and then the remaining culture was infected with ICP1_2006_E ΔCRISPR Δ*cas2-3* at a multiplicity of infection (MOI) of 2.5 for 20 min at 37°C. The uninfected and infected samples were then boiled for 10 min, and 2 μL was used as a template for PCR using primers to detect the circularized PLE listed in Table S1. Replication of PLE upon ICP1 infection was measured as previously described (18, 20). Briefly, 2-mL cultures of V. cholerae were grown to an OD_600_ of ∼0.3 before being infected with ICP1_2006_E ΔCRISPR Δ*cas2-3* (or the Δ*helA* derivative) at an MOI of 2.5. Immediately before the phage addition, 100 μL of culture was boiled for the T0 sample. Cultures were returned to the incubator. After 30 min, 100 μL of infected culture was removed and boiled for the T30 sample. Serial dilutions of boiled samples were then used as the template for quantitative PCR (primers are listed in Table S1), and fold change in replication was determined as the amount of DNA in the T30 sample relative to the T0 sample. All samples were run in biological triplicates and technical duplicates. Lysis kinetics were determined as previously described (22). Briefly, V. cholerae strains were grown to an OD_600_ of 0.3 in 2-mL cultures. Culture (150 μL) was then added to a 96-well plate containing phage. Kinetics were then recorded by a SpectraMax i3x (Molecular Devices) via measurements of the OD_600_ every 2 min. Over the course of the run, the plate was incubated at 37°C and shaken for 1 min between reads. PLE transduction was performed with magnesium as previously described (21).

### Phage spot assays.

Mid-log-phase V. cholerae was added to 0.5% molten LB agar, poured on a solid agar plate, and allowed to solidify. Tenfold dilutions of phage were overlaid in 3-μL spots. After spots were dry, plates were incubated at 37°C for ∼6 h prior to imaging. Images are representative of three independent experiments.

### V. cholerae phylogeny.

A cladogram of 148 Vibrio cholerae isolates was constructed based on 532 single nucleotide variations (SNVs). These variable locations were determined by aligning the assembled sequence of each isolate to the reference strain N16961 using MUMmer (v. 3.0). Only nucleotide locations that were present in all 148 isolates as well as the reference were considered for analysis. The binary matrix of SNVs was clustered with R using R’s hclust package (v. 1.2.3). The SXT ICE present in each isolate was determined previously by whole-genome sequencing (24).

### Quantification and statistical analysis.

For qPCR and transduction assays, data from each independent biological replicate is shown, and the height of the bar indicates the average. For lysis curves, the shaded area indicates the standard deviation of the average fold change from three independent biological replicates. Spot plates and agarose gels are representative of at least three independent experiments.
