# Supplementary material for: Evolutionary Sweeps of Subviral Parasites and Their Phage Host Bring Unique Parasite Variants and Disappearance of a Phage CRISPR-Cas System
Source: mBio. 2022 Feb 15;13(1):e03088-21. doi: 10.1128/mbio.03088-21 (PMC8844924; doi:10.1128/mbio.03088-21)
Supplement: TABLE S1 [file mbio.03088-21-st001.docx]

**TABLE S1**

| REAGENT or RESOURCE | SOURCE | IDENTIFIER |
| --- | --- | --- |
| **Bacterial and Virus Strains** | | |
| *V. cholerae* E7946 (used as PLE(-) Figure 3) | Laboratory collection | KDS6 |
| *V. cholerae* E7946 with PLE1 (used as PLE1 in Figure 3) | O’Hara et al., 2017 | KDS36 |
| *V. cholerae* clinical isolate with PLE10 | LeGault et al., 2021 | BFS87 |
| *V. cholerae* clinical isolate with PLE10 marked with kanamycin resistance cassette for transduction. | This paper | KDS311 |
| *V. cholerae* E7946 with PLE10 (used as PLE10 in Figure 3) | This paper | KDS310 |
| ICP1_2006_E ΔCRISPR Δ*cas2-3* (referred to as ICP1^2006^ in Figure 3 legend) | McKitterick and Seed, 2018 | KSΦ38 |
| ICP1_2006_E ΔCRISPR Δ*cas2-3* Δ*helA* (referred to as Δ*helA* in Figure 3 legend) | McKitterick et al., 2019 | ACMΦ268 |
| ICP1 2019_Dha_G (accession number MW794185; used as *odn*(+) phage in Figure 3F) | LeGault et al., 2021 | PFS233 |
| ICP1 2019_Dha_G Δ*odn* (used as *odn*(-) phage in Figure 3F) | This paper | KSΦ157 |
| ICP1 2017_Dha_AB (accession number MW794154; used as CRISPR(+) phage in Figure 3F). | LeGault et al., 2021 | PFS229 |
| ICP1 2017_Dha_AB Δ*cas2-3* (used as CRISPR(-) phage in Figure 3F). | This paper | KSΦ158 |
| ICP1 2018_Mat_159 (accession number  MW794177 referred to as 159 in Figure 4C) | LeGault et al., 2021 | PFS159 |
| ICP1 2018_Mat_160 (accession number  MW794178 referred to as 160 in Figure 4C) | LeGault et al., 2021 | PFS160 |
| ICP1 2018_Mat_164 (accession number  MW794179 referred to as 164 in Figure 4C) | LeGault et al., 2021 | PFS164 |
| ICP1 2018_Mat_166 (accession number  MW794180 referred to as 166 in Figure 4C) | LeGault et al., 2021 | PFS166 |
| ICP1 2018_Mat_167 (accession number  MW794181 referred to as 167 in Figure 4C) | LeGault et al., 2021 | PFS167 |
| ICP1 2018_Mat_170 (accession number  MW794182 referred to as 170 in Figure 4C) | LeGault et al., 2021 | PFS170 |
| **Oligonucleotides** | | |
| Primer to detect ICP1-encoded CRISPR:  CGGAGAAATTCAACAGTTATGG | This paper | KS252 |
| Primer to detect ICP1-encoded CRISPR:  CAGAAAGTATTGCGGCTAGG | This paper | KS253 |
| Primer to detect ICP1-encoded *odn*: CCACATATTGTTTCCTCCAATAATTTCATATCC | This paper | KS1085 |
| Primer to detect ICP1-encoded *odn*:  CTATGACCATGATTACGCCACATAGACGACCACCTGCAAC | This paper | KS1086 |
| Primer to detect PLE1 and PLE10 circularization: GCTACTCTCCGTTAAATTCCG | This paper | KS365 |
| PLE1 and PLE10 circularization primer: CCGCTATCTTTCGAGGTAGC | This paper | KS364 |
| PLE qPCR primer:  GTCGGTTTCTTCCGATAAGG | This study | MN413 |
| PLE qPCR primer:  GTTTGAATATCCCACGAGCC | This study | MN414 |
| PLE1 screening primer: GGTGATTGTCCTTTCATTTGGG | This study | ACM349 |
| PLE1 screening primer: GTGATGCTGTTCAAACAGGG | This study | ACM350 |
| **Recombinant DNA** | | |
| Plasmid: Ptac EV | McKitterick et al., 2019 | ACM707 |
| Plasmid: P*_tac_*-*helA* | McKitterick et al., 2019 | ACM709 |
| Plasmid: P*_tac_*-*helB* | McKitterick et al., 2019 | ACM711 |

**References:**

**O’Hara BJ**, **Barth ZK**, **McKitterick AC**, **Seed KD**. 2017. PLoS Genet **13**:e1006838

**LeGault KN**, **Hays SG**, **Angermeyer A**, **McKitterick AC**, **Johura F-T**, **Sultana M**, **Ahmed T**, **Alam M**, **Seed KD**. 2021. Science **373** :eabg2166.

**McKitterick AC**, **Seed KD**. 2018. Nature Communications **9**:2348–8.

**McKitterick AC**, **Hays SG**, **Johura F-T**, **Alam M**, **Seed KD**. 2019. Cell Host & Microbe **26**:504–514.e4.
