# Supplementary figures and images for: Evolutionary Sweeps of Subviral Parasites and Their Phage Host Bring Unique Parasite Variants and Disappearance of a Phage CRISPR-Cas System
Source: mBio. 2022 Feb 15;13(1):e03088-21. doi: 10.1128/mbio.03088-21 (PMC8844924; doi:10.1128/mbio.03088-21)

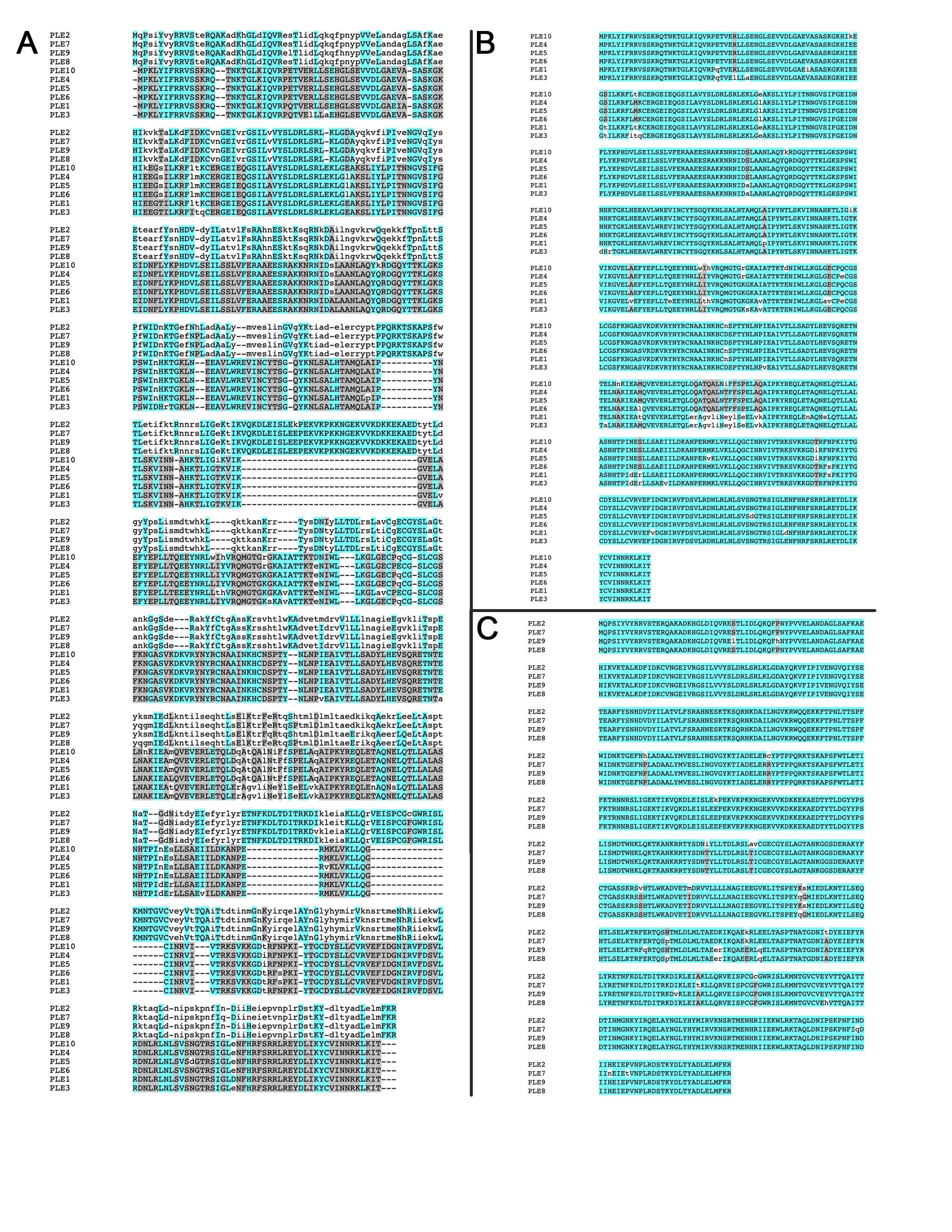

Supplement: FIG S2 [file mbio.03088-21-sf002.jpg]

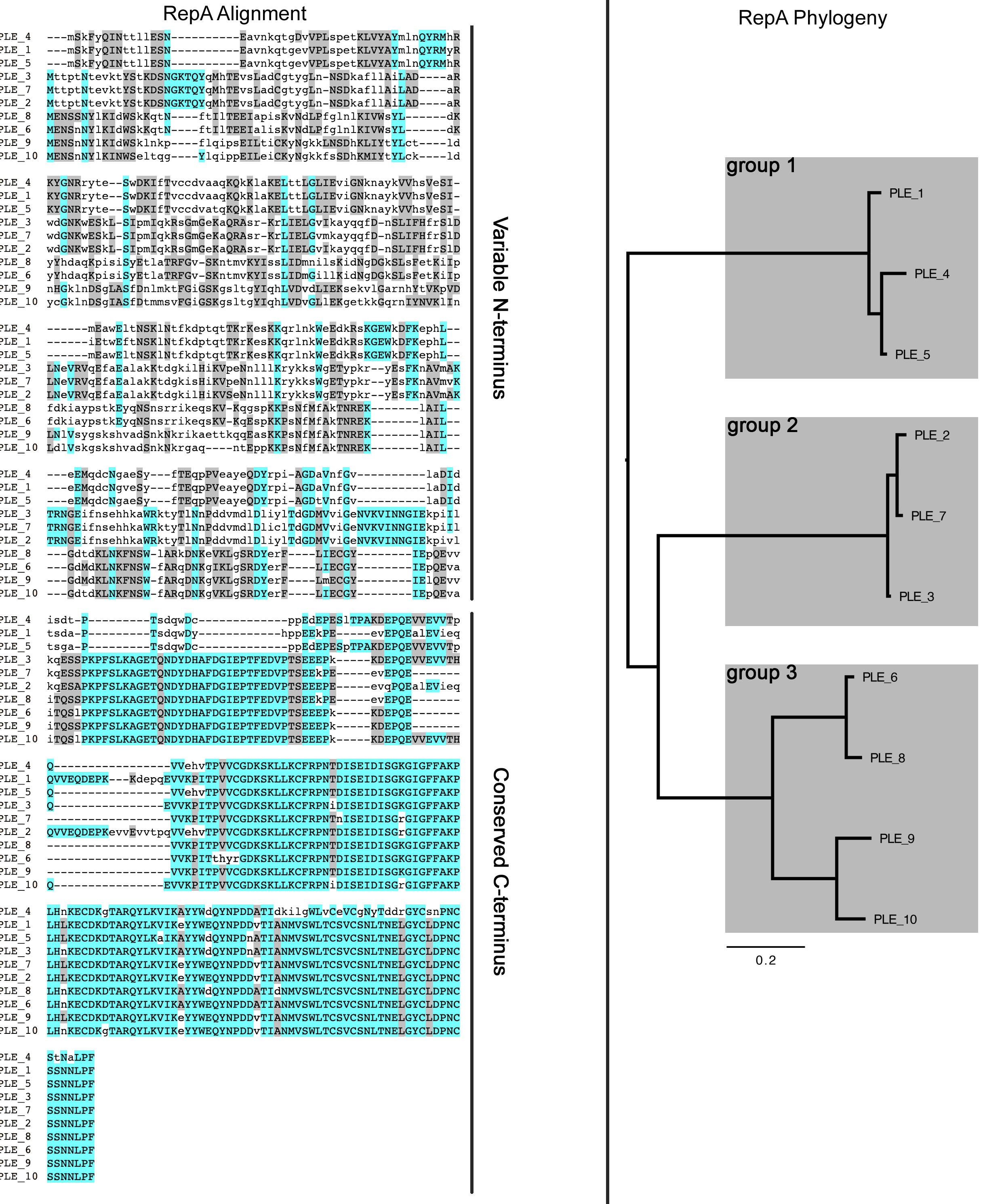

Supplement: FIG S4 [file mbio.03088-21-sf004.jpg]
